# Reniform Nematode Management Using Winter Crop Rotation and Residue Incorporation Methods in Greenhouse Experiments

**DOI:** 10.2478/jofnem-2023-0035

**Published:** 2023-09-13

**Authors:** Rebeca Sandoval-Ruiz, Zane J. Grabau

**Affiliations:** Entomology and Nematology Department, University of Florida 1881 Natural Area Drive, Gainesville, FL 32611, United States

**Keywords:** *Avena sativa*, *Brassica carinata*, biofumigation, carinata, cotton, *Gossypium hirsutum*, hairy vetch, host, management, oat, reniform nematode, rotation, *Rotylenchulus reniformis*, *Vicia villosa*, winter crops

## Abstract

*Rotylenchulus reniformis* (reniform nematode, RN) is an important pathogen in cotton production. Cultural practices such as crop rotation and biofumigation—management of soil pathogens by biocidal compounds from crop residues—may help manage RN. The objective of this study was to evaluate the efficacy of winter crops for RN management through combinations of rotation and crop residue incorporation in a cotton greenhouse experiment. A total of 10 treatments were evaluated in soil inoculated with RN: three winter crops (carinata, oat, or hairy vetch) grown in rotation with no shoot organic matter (OM) incorporated (1–3), fresh shoot OM incorporated (4–6), or dry shoot OM incorporated (7–9), and a fallow control (10). Roots were re-incorporated in all treatments except fallow. Subsequently, cotton was grown. Oat and fallow were better rotation crops to lower soil RN abundances at winter crop termination than hairy vetch and carinata. After the OM incorporation treatments and cotton growth, oat was generally more effective at managing RN in cotton than carinata or hairy vetch. Within each crop, incorporation treatment generally did not affect RN management. Cotton growth was not consistently affected by the treatments.

Reniform nematode (RN), *Rotylenchulus reniformis*, is an important pathogen that limits cotton (Gossypium hirsutum) yield in the United States (U.S.) (Robinson, 2007). It alters cotton growth and quality (Koebernick et al., 2021). Economic damage caused by RN on cotton in the U.S. leads to an annual yield loss of 0.1–5% (Lawrence, 2021). The use of chemical nematicides can improve cotton yield but not enough to equal the income of a farm without RN (Lawrence, 2021). Reniform nematode-resistant cotton cultivars are available but not yet widely implemented (Turner et al., 2023). Therefore, cultural practices such as crop rotation could help to mitigate damage from RN if used as part of integrated nematode management strategies.

Crop rotation with non-host crops can help to reduce soil nematode populations by disrupting their life cycles (Cherr et al., 2006). Moreover, the use of poor or non-host winter crops can reduce the nematode population before the summer cash crop is established (Acharya et al., 2021). Winter crops that may be rotated with cotton in the Southeastern region, such as brassicas, cereals, and legumes, vary in their host status to RN. *Avena sativa* (oat) is a cereal that is a poor RN host (Asmus et al., 2008). In contrast, *Vicia villosa* subsp. *villosa* (hairy vetch) is a leguminous crop classified as a good RN host (McSorley and Dickson, 1989; Jones et al., 2006).

However, winter cover cropping is not widely adopted in cotton or other agronomic cropping systems in the Southeast. For example, cover crop use across cropland in Florida was estimated at 1.5% in 2017 (USDA-NASS, 2019). Small grains, such as oat, are among the most common cover crops in the region. Cover crops like oat have benefits such as improving soil fertility (Zhao et al., 2010), outcompeting weeds (Wang et al., 2004), and managing certain nematodes (Wang et al., 2004; Asmus et al., 2008). Growers are not able to sell these winter cover crops for revenue though, which may be one factor limiting adoption.

In contrast, *Brassica carinata* (carinata) is an emerging biofuel cash crop that is grown in the winter in the Southeast. Carinata offers the prospect to farm more than 1.4 million hectares that are typically left fallow during winter and may be incorporated into cropping systems without competing with food production from summer cash crops (Seepaul et al., 2021). Carinata was recently described as a poor RN host (Sandoval-Ruiz and Grabau, 2023). Other properties of carinata, including a highly developed root system and high content of glucosinolates—compounds toxic to many organisms—make this crop of interest for management of RN and other nematodes (Barro and Martin, 1999; Lal et al., 2019).

When toxic compounds, such as glucosinolates from brassicas, are released from crop residues that have been incorporated in the soil, this can help manage soil-borne pathogens in a process known as biofumigation (Kirkegaard et al., 1993). Crops produce these toxic compounds to protect themselves from pathogens, such as nematodes (Zaynab et al., 2019). The incorporation of winter crop residues after crop harvest or termination is a frequent practice. Therefore, if winter crops with secondary metabolites toxic to nematodes are grown, nematode management could be improved beyond non-host benefits. Aside from glucosinolates produced by carinata (Hagos et al., 2020), the following compounds produced by winter crops have demonstrated nematicidal effects: avenacins produced by oat (Mylona et al., 2008), and cyanides produced by hairy vetch (Kamo et al., 2006). For example, dry OM from oat can suppress the incidence of *Meloidogyne* (Akhtar and Alam, 1993), and incorporating oat residues into soil at 0.2% w/w rate reduced RN reproduction compared to a control without oat application in a greenhouse study (Gardiano et al., 2013). Dry OM from hairy vetch reduced RN reproduction when applied at 0.2% w/w under greenhouse conditions (Gardiano et al., 2013).

Methods for handling residues from winter crops following harvest or termination vary and may affect RN management. Residues of winter crops can be harvested without incorporating the residues or can be incorporated as fresh material into the soil, known as green manure (Larkin, 2013). Green manure from brassicaceous crops, cereals, and legumes has been used to manage plant parasitic nematodes (Crow et al., 1996; Collange et al., 2011; Dutta et al. 2019). However, in carinata production, the plant is left to dry in the field after harvest and the dry carinata residues are subsequently incorporated into the soil. Therefore, the assessment of winter crop fresh and dry OM residues is important in terms of practical management.

The combined effects of carinata rotation and residue incorporation for managing RN have not been previously studied. This information is important for helping farmers make informed decisions about adopting carinata since RN is an important pest in the region. Evaluation of the same information in other common Southeast winter cover crops could guide strategies for handling winter crop residues to manage RN. Therefore, the objective of this study was to evaluate the efficacy of rotation with winter crops in combination with various residue incorporation strategies for managing RN in cotton in greenhouse experiments.

## Materials and Methods

### Location and experimental design

**Figure 1:**
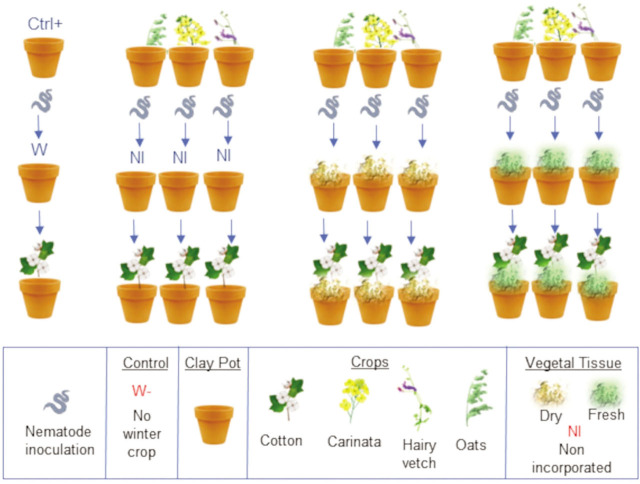
Graphical representation of the methodology to evaluate rotation of winter crops—with varying crop residue incorporation methods—to manage reniform nematode and cotton plant growth.

The objectives were investigated in a repeated pot experiment conducted in a polycarbonate greenhouse at the University of Florida Entomology and Nematology Department in Gainesville, FL. Two trials, Trial 1 and Trial 2, were performed in a completely randomized design, with six replicates that utilized winter crops and method of crop residue incorporation as the only factors. A total of 10 treatments (Fig. 1) were evaluated: three winter crops (carinata, oat, or hairy vetch) grown in rotation with 1) no shoot OM incorporated (1–3), 2) fresh shoot OM incorporated (4–6), or 3) dry shoot OM incorporated (7–9); and a fallow control (10). This experiment simulated crop rotation without shoot incorporation (harvested), incorporating fresh residue, and incorporating dried residue. For each treatment, fresh roots were reincorporated because this mimics field production practices. Crops evaluated were the major variety of carinata available at the time of experiment establishment, ‘Avanza 641’ (Nuseed Co., Sacramento, CA), and unknown varieties of hairy vetch and oat.

### Inoculum and soil preparation

The RN population for this research came from a naturally infested field in Tift County, Georgia, and was maintained in cotton plants in greenhouse conditions. The inoculum was obtained based on Hussey and Barker (1973), as explained in Sandoval and Grabau (2023). Soil used in this experiment was autoclaved at 121 °C for 90 minutes in an Amsco Lab (OH, USA) 250 LV autoclave. The soil was a Chipley-Foxworth-Albany complex (91% sand, 6.8% silt, and 2.4% clay with 1.7% OM) from the University of Florida North Florida Research and Education Center–Suwannee Valley near Live Oak, FL.

### Trial establishment

The experiment had three stages: 1) establishment, 2) winter crop residue application, and 3) evaluation (Fig. 1). During the first stage, winter crops were planted in clay pots (15 cm top diameter) containing 1000 cm^3^ of autoclaved soil that was mixed in a plastic bag with 3500 RN (eggs and juveniles). Nematodes were inoculated into each pot on the same day as inoculum extraction. In the second stage, at winter crop termination, the entire plant was removed from the pot. For all treatments, the roots were cut into pieces smaller than 0.5 cm and were reincorporated into the same pot with the original soil from the first stage. The plant shoot was discarded for treatments without OM incorporation. For the dry OM treatments, after harvesting the winter plants, the shoot was placed in paper bags in an oven (SPX, Blue M Electric Co, Blue Island, IL) for three days at 60°C (140° F). For the dry OM and fresh OM treatments, the dry or fresh shoots of the winter crops were ground into small particles in a domestic coffee grinder. Subsequently, the OM was mixed in a plastic bag with the soil from the same pot as the first stage of the experiment. Pots were placed in the greenhouse and after 12 days, cotton seeds (‘Deltapine 1646B2XF’, Bayer CropScience, St. Louis, MO) were planted directly into the soil and left to grow until harvest, two months later. Information about trial schedules is provided in Table 1.

**Table 1.** Schedule for plant data collection and management in greenhouse trials.

	**Trial 1**		**Trial 2**	
	
	**Date**	**DAP^a^**	**Date**	**DAP^a^**
1. Winter Crops				
1.1 Planting and nematode inoculation	12 November 2021	0	29 December 2022	0
1.2 Harvest				
• Dry OM treatments	25 January 2022	74	14 March 2022	75
• No OM treatments	27 January 2022	76	16 March 2022	76
• Fresh OM treatments	27 January 2022	76	16 March 2022	76
1.3 OM incorporation	28 January 2022	77	17 March 2022	78
2. Cotton				
2.1 Planting	8 February 2022	0	28 March 2022	0
2.2 Plant growth assessment	6 April 2022	57	24 May 2022	57
2.2 Harvest	10 April 2022	61	27 May 2022	60

a Days after planting.

### Plant densities

The final plant densities of each crop were based on recommended planting density for field production as adapted to 15-cm-diameter pots as measured at the top of the pot (area= 176.7 cm^2^). Therefore, the final density for the first stage was six oat, two carinata, two canola, or one hairy vetch per pot (Seepaul et al., 2019; Harker et al., 2015; Forsberg and Reeves, 1995; Bamford and Entz, 2016). Winter crops were initially seeded at double the recommended density, but one week after planting, pots were thinned to the final density specified for each crop. Cotton in the second stage of the experiment was also seeded at double the recommended planted density but 5 days after planting was thinned to the recommended density of 2 plants per pot.

### Trial maintenance

The soil temperature was measured using a HOBO MX TidbiT 400 (Onset Computer Corporation, MA, USA). The average temperature while the winter crops were growing and incorporated was 19.6°C (max=36.8°C, min=6.3°C) for Trial 1, and 21.4°C (max=48.8°C, min=8.0°C) for Trial 2. The temperature when the cotton plants were growing was 23.4°C (max=46.0°C, min=8.2°C) for Trial 1, and 25.6°C (max=46.0°C, min=11.0°C) for Trial 2. No supplemental light or fertilizer was used, and plants from all treatments were watered daily by hand.

### Nematode sampling and quantification

Subsamples of 100 cm^3^ soil for nematode extraction were taken after winter crops and cotton were harvested. Nematodes were also extracted from the entire cotton root system at harvest and cotton root weight was measured to calculate RN density on roots. Nematode extractions from roots were done using the same method for the inoculum preparation as previously explained, while the extraction from soil was done by the sucrose centrifugation method (Jenkins, 1964). Nematode counting and identification were done using a 400X inverted Primovert microscope (Carl Zeiss Inc, Thornwood, NY). Reniform nematode soil abundances (vermiform nematodes) at winter crop and cotton termination, RN population densities (eggs and vermiform nematodes) per gram of roots, and reproduction factor (RF, RF= final population/initial population, final population= RN eggs and vermiform in soil and roots) (Seinhorst, 1967) are reported.

### Plant growth assessment

Plant growth assessments were made at termination of winter crops and cotton. For winter crops, the total fresh weight of crop residues to be incorporated for each respective treatment was measured. Only root weight was measured for rotation with no OM incorporation treatments, but root and shoot fresh weights were measured for rotation with fresh OM or dry OM treatments. Additionally, the total OM rate applied per pot (%w/w) was calculated for each treatment ((∑OM matter applied*100)/1500 g soil). For the dry OM treatments, the ∑OM matter applied = dry shoot weight+ fresh root weight. For the fresh treatments: ∑OM= fresh shoot weight + fresh root weight. For the no OM treatments, ∑OM= fresh root weight. For this experiment, 1500 g soil was equal to 1000 cm^3^ soil. The variables measured for cotton plants were height, root fresh weight, shoot fresh and dry weight, Soil Plant Analysis Development (SPAD), and canopy cover. SPAD values were taken on the third fully expanded leaf from the apex using a SPAD-502 chlorophyll meter (Konica Minolta Sensing Inc., Osaka, Japan). To assess the canopy cover, each pot was placed in the center of a 1 m^2^ white cardboard, and photos were taken at 1 m height, parallel to the top of the pot. Pictures were cut to only show 1 m^2^ using the computer imaging program GIMP (GNU Image Manipulation Program) 2.10.20 (The GIMP Team, Berkley, CA) and were evaluated using Canopeo (Patrignani and Ochsner, 2015).

### Statistical analysis

Data analysis was done in RStudio version 2021.09.0 (The R Foundation for Statistical Computing, Vienna, Austria). To ensure ANOVA model assumptions were met, normality was assessed by the Shapiro-Wilk test, and homogeneity of variances by Levene's test. For nematode and plant parameters, trials were analyzed separately because of trial-by-treatment interactions (ANOVA, *P* ≤ 0.05). Plant parameters that did not meet the statistical assumptions were square root or ln(x+1) transformed to meet them. The % w/w OM applied per pot for the rotation treatments with fresh or dry OM incorporation and the plant parameters were analyzed by one-way ANOVA. For the winter crops and cotton, the plant parameters with significant treatment effects (ANOVA, *P* ≤ 0.05) were analyzed by Tukey's honesty significant test (HSD). Nematode variables were analyzed by Kruskal-Wallis non-parametric test because variables did not meet ANOVA model assumptions. For nematode variables, means were separated by multiple comparisons with Bonferroni's method at a 5% significance level, if the Kruskal-Wallis's test was significant (*P* < 0.05). Nematode soil populations at winter crop termination were analyzed by crop only because crop-OM incorporation treatment combinations had not been implemented yet. Data is displayed as boxplots in the figures. The black center line represents the median (50^th^ percentile), the lower limit of boxes symbolizes the 25^th^ percentile, the upper limit of boxes the 75^th^ percentile, whiskers indicate the 5^th^ and 95^th^ percentiles, and outliers denote values beyond the upper and lower whiskers and are indicated as black dots.

## Results

### Reniform nematode soil abundances

**Figure 2:**
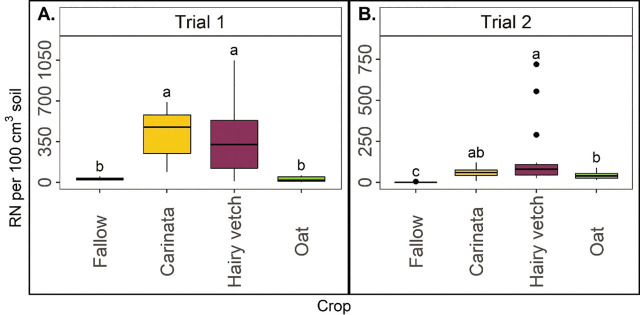
Reniform nematode (RN) in 100 cm^3^ of soil at winter crop termination in Trial 1 (A) and Trial 2 (B). Letters indicate treatment separation by Kruskal-Wallis with Bonferroni's method, *P* < 0.05. Data is displayed as boxplots.

Winter crops are significantly affected by RN abundance in the soil at winter crop termination. The abundance of RN in 100 cm^3^ soil was higher in carinata and hairy vetch than in fallow and oat in Trial 1 (Fig. 2A). In Trial 2, consistent with Trial 1, hairy vetch had greater RN abundance than fallow and oat, but in this trial, carinata had intermediate values (Fig. 2B).

**Figure 3:**
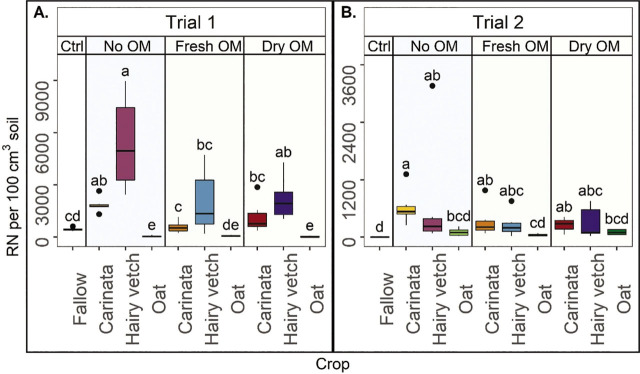
Reniform nematode (RN) in 100 cm^3^ of soil at cotton harvest, by crop and organic matter incorporation method, in Trial 1 (A) and Trial 2 (B). Ctrl= fallow control, No OM= rotation with no shoot organic matter (OM) incorporated. Fresh OM= treatments with fresh shoot OM, Dry OM= treatments with dry shoot OM. Fresh roots were incorporated in each treatment. Letters indicate treatment separation by Kruskal-Wallis with Bonferroni's method, *P* < 0.05. Data is displayed as boxplots.

Crops significantly affected RN abundance in the soil at cotton termination, but the incorporation type did not have a consistent effect. In general, oat and fallow managed final RN soil abundances better than carinata and hairy vetch. In Trial 1, oat no OM and dry OM had lower soil RN abundance relative to fallow (Fig. 3A). No OM from carinata, and no OM and dry OM from hairy vetch had greater RN abundances than fallow, and all other treatments were similar to fallow. In Trial 1, RN soil abundance did not vary by incorporation type in oat, but carinata and hairy vetch did have greater RN soil abundance for no OM than fresh OM and intermediate abundance for dry OM (Fig. 3A). In Trial 2, all of the treatments had similar or greater soil RN abundance relative to fallow (Fig. 3B). The oat treatments, for all incorporation types, were not different from fallow. However, in Trial 2, all carinata and hairy vetch treatments increased RN abundance relative to fallow (Fig. 3B). In Trial 2, there were no differences in the final RN soil abundance among OM incorporation types within each crop (Fig. 3B).

### Reniform nematode root abundances from cotton

**Figure 4:**
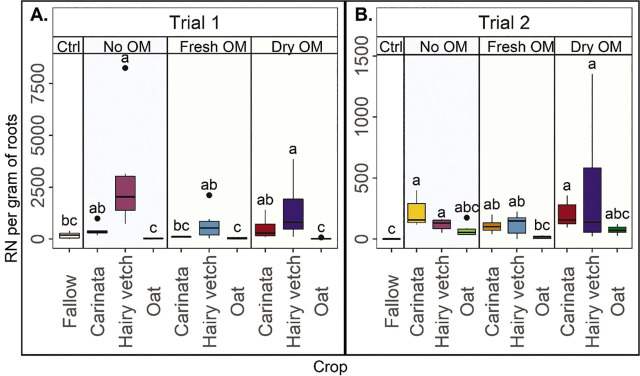
Reniform nematodes (RN) from cotton roots as affected by crop and organic matter incorporation treatments. Reniform nematode abundances are expressed as total RN (eggs plus vermiform nematodes) per gram of cotton roots in Trial 1 (A) and Trial 2 (B) Ctrl= fallow control, No OM= rotation with no shoot organic matter (OM) incorporated. Fresh OM= treatments with fresh shoot OM, Dry OM= treatments with dry shoot OM. Fresh roots were incorporated in each treatment. Letters indicate treatment separation by Kruskal-Wallis with Bonferroni's method, *P* < 0.05. Data is displayed as boxplots.

In general, RN root abundances varied by crop but did not consistently vary by incorporation treatment (Fig. 4). Consistently in both trials, fresh OM from oat was the most effective treatment to manage RN root abundances and no OM and dry OM from hairy vetch were the least effective. In Trial 1, RN root abundances were generally lower in the oat treatments, greater in the no OM and dry OM from hairy vetch, and intermediate in the other treatments (Fig. 4A). The no OM and dry OM from hairy vetch had greater RN root abundance than fallow, and all other treatments were statistically similar to fallow (Fig. 4A). In Trial 2, the fresh OM from oat and fallow had generally lower RN abundances in the roots, while the no OM and dry OM from carinata and hairy vetch had the greatest abundances, and all the other treatments had intermediate values (Fig. 4B). In this trial, none of the oat treatments were different from fallow but all the carinata and hairy vetch treatments had greater RN abundances than fallow (Fig. 4B). In both trials, there were no differences in RN root abundances among OM incorporation types within each crop (Fig. 4).

### Reniform nematode reproduction factor in cotton

**Figure 5:**
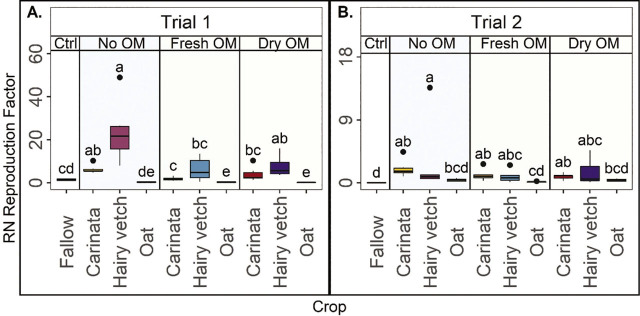
Reniform nematode (RN) reproduction factor (total RN per pot final population/inoculated initial population), by crop, at cotton harvest in Trial 1 (A) and Trial 2 (B). Ctrl= fallow control, No OM= rotation with no shoot organic matter (OM) incorporated. Fresh OM= treatments with fresh shoot OM, Dry OM= treatments with dry shoot OM. Fresh roots were incorporated in each treatment. Letters indicate treatment separation by Kruskal-Wallis with Bonferroni's method, *P* < 0.05. Data is displayed as boxplots.

Crops significantly impacted the RN reproduction factor (RF), but incorporation treatment did not consistently affect RF (Fig. 5). Oat and fallow were generally good whereas hairy vetch no OM was generally poor for managing RN based on RF, with other treatments intermediate or variable. In Trial 1, the fresh OM and dry OM from oat were the only treatments that reduced RF relative to fallow (Fig. 5A). In Trial 1, RF did not vary by incorporation treatment within oat, but within carinata and hairy vetch, RF was greater with no OM than fresh OM and intermediate for dry OM (Fig. 5B). In Trial 2, RF was less for fallow than most treatments and the oat treatments had RF that, statistically, was similarly as low as fallow. In this trial, none of the treatments reduced RF compared to fallow, and any of the treatments from carinata and hairy vetch had greater RF than fallow (Fig. 5B). In this trial, the incorporation type, within crop, did not have any influence on the RF (Fig. 5B).

### Winter crop plant parameters

In Trial 1, the fresh weight of residues was greater for oat than hairy vetch or carinata independently of the incorporation treatment but in Trial 2 there was more variation among treatments (Table 2). In Trial 2, oat without OM had greater fresh residue weight than any dry OM treatment or fresh hairy vetch (Table 2). The final OM rate applied per pot varied by treatment (Table 2). In Trial 1, OM rate was greater for oat than any other crop, regardless of incorporation type (Table 2). In Trial 2, the OM rate applied per pot was greater for fresh oat and carinata than most other treatments (Table 2).

**Table 2.** Winter crop residue measurements by crop rotation and residue incorporation method.

**Treatment**	**Trial 1^a^**	

	**Fresh weight (g) of residues added^b^**	**Final OM added (%w/w)^c^**
*p*-value	***	***
Rotation+No OM^d^		
Carinata	20.3 ± 1.6 b	1.3 ± 0.1 bc
Hairy vetch	27.0 ± 5.2 b	1.8 ± 0.3 b
Oat	68.4 ± 10.9 a	4.5 ± 0.7 a
Rotation+Fresh OM		
Carinata	37.8 ± 3.0 b	2.5 ± 0.2 b
Hairy vetch	26.0 ± 7.5 b	1.7 ± 0.5 b
Oat	78.5 ± 5.4 a	5.2 ± 0.3 a
Rotation+Dry OM		
Carinata	28.4 ± 2.5 b	0.9 ± 0.0 bc
Hairy vetch	26.9 ± 3.4 b	1.4 ± 0.1 bc
Oat	91.1 ± 2.7 a	4.2 ± 0.3 a

**Treatment**	**Trial 2**	

	**Fresh weight (g) of residues added**	**Final OM added (%w/w)**
*p*-value	***	***
Rotation+No OM		
Carinata	27.9 ± 2.2 abc	0.6 ± 0.0 ef
Hairy vetch	27.3 ± 7.3 abc	1.4 ± 0.4 cd
Oat	44.7 ± 4.8 a	1.9 ± 0.1 bc
Rotation+Fresh OM		
Carinata	34.4 ± 3.1 abc	2.2 ± 0.2 ab
Hairy vetch	22.7 ± 4.2 c	1.5 ± 0.2 cd
Oat	42.9 ± 5.5 ab	2.8 ± 0.3 a
Rotation+Dry OM		
Carinata	23.8 ± 2.9 bc	0.6 ± 0.1 ef
Hairy vetch	15.2 ± 2.9 c	1.1 ± 0.2 de
Oat	21.2 ± 2.9 c	1.5 ± 0.2 cd

a Mean ± standard error (n=6). Letters within the same plant parameter (column) indicate significant differences among the treatments by trial (Tukey HSD, α=0.05) with ANOVA “***”= *P* ≤ 0.01.

b Fresh weight for Rotation+No OM=root fresh weight, for Rotation+ Fresh OM and Rotation+dry OM=root fresh weight+shoot fresh weight.

c Final OM added (%w/w) is root fresh weight for Rotation+No OM, fresh root weight+fresh shoot weight for Rotation+Fresh OM, and fresh root weight+dry shoot weight for Rotation+Dry OM.

d Rotation+No OM, Rotation+Fresh OM, and Rotation+Dry OM are rotation with no incorporation of shoot organic matter, incorporation of fresh organic matter, and incorporation of dry organic matter, respectively. Fresh roots were incorporated for each treatment.

### Cotton plant parameters

In Trial 1, all plant parameters, except the shoot dry weight, were affected by treatments (Table 3). In Trial 2, the fresh shoot weight was the only plant parameter affected by treatments (Table 3). Therefore, in general, there were no consistent treatment effects on plant growth for the two trials (Table 3). In Trial 1, the canopy cover, height, and shoot fresh weight were each greater for carinata no OM and fresh OM than for the oat fresh OM. Cotton height and shoot fresh weight were also greater following carinata no OM and fresh OM than oat dry OM (Table 3). The root fresh weight did not have consistent results between trials (Table 3). In Trial 1, the root fresh weight of the cotton plants was greater following all oat treatments compared to the dry and fresh OM from hairy vetch and the fallow treatment, with all the other treatments intermediate (Table 3). In Trial 2, carinata fresh OM and hairy vetch no OM had greater root fresh weight than carinata dry OM and fallow; all other treatments had intermediate values (Table 3). In Trial 1, SPAD was greater following fallow than carinata no OM and hairy vetch treatments (Table 3). Additionally, oat no OM had a greater SPAD value than the dry or no OM treatment from hairy vetch. There were no differences in SPAD among the other treatments (Table 3).

**Table 3.** Influence of winter crop rotation and residue incorporation method, on cotton growth and SPAD value in greenhouse conditions under the presence of reniform nematode.

**Treatment**	**Canopy cover**	**Height (cm)**	**Root fresh weight (g)**	**Root fresh weight (g)**	**Shoot fresh weight (g)**	**SPAD**

	**Trial 1^a^**					
*p*-value^b^	**	***	***	ns	***	***
Control - (Fallow)	4.0 ± 0.3 abc	10.8 ± 0.4 ab	4.6 ± 0.4 e	1.4 ± 0.1	4.7 ± 0.3 ab	46.1 ± 0.9 a
Rotation+No OM^c^						
Carinata	5.2 ± 0.3 a	12.0 ± 0.5 a	13.3 ± 1.0 abc	1.3 ± 0	5.3 ± 0.3 a	38.8 ± 1.3 bc
Hairy vetch	4.0 ± 0.3 abc	10.7 ± 0.3 ab	11.1 ± 0.9 bcd	1.0 ± 0	4.3 ± 0.3 ab	36.6 ± 1.1 c
Oat	4.0 ± 0.5 abc	10.4 ± 0.6 ab	20.5 ± 2.7 a	1.0 ± 0	3.8 ± 0.3 ab	44.8 ± 1.2 ab
Rotation+Fresh OM						
Carinata	4.9 ± 0.4 ab	11.8 ± 0.5 a	11.0 ± 1.9 bcd	1.3 ± 0.1	5.3 ± 0.4 a	41.6 ± 0.7 abc
Hairy vetch	3.9 ± 0.5 abc	11.0 ± 0.7 ab	6.3 ± 0.7 de	1.1 ± 0.2	4.1 ± 0.6 ab	38.2 ± 1.6 bc
Oat	2.9 ± 0.2 c	9.2 ± 0.5 b	14.5 ± 2.1 ab	0.9 ± 0.0	3.3 ± 0.2 b	42.1 ± 1.3 abc
Rotation+Dry OM						
Carinata	3.8 ± 0.3 abc	10.0 ± 0.4 ab	7.4 ± 0.6 cde	1.0 ± 0.0	4.0 ± 0.2 ab	40.5 ± 1.4 abc
Hairy vetch	3.9 ± 0.4 abc	10.4 ± 0.6 ab	4.7 ± 0.8 e	0.9 ± 0.0	3.8 ± 0.3 ab	37.9 ± 1.7 c
Oat	3.2 ± 0.3 bc	9.1 ± 0.3 b	17.2 ± 2.2 ab	0.9 ± 0.0	3.2 ± 0.2 b	41.1 ±1.9 abc

**Trial 2^a^**						

*p*-value^b^	ns	ns	***	ns	ns	ns
Control - (Fallow)	5.5 ± 0.5	12.0 ± 0.6	4.0 ± 0.3 b	1.6 ± 0.1	5.2 ± 0.4	56.2 ± 1.3
Rotation+No OM						
Carinata	8.2 ± 1.7	14.0 ± 1.1	6.5 ± 0.6 ab	1.9 ± 0.1	6.8 ± 0.6	49.9 ± 0.7
Hairy vetch	8.9 ± 2.7	16.5 ± 2.1	8.3 ± 1.4 a	2.1 ± 0.3	6.8 ±1.2	47.8 ± 3.4
Oat	6.9 ± 0.8	13.5 ± 0.8	6.1 ± 0.6 ab	1.7 ± 0.1	5.4 ± 0.4	49.6 ± 1.5
Rotation+Fresh OM						
Carinata	9.8 ± 1.7	15.4 ± 1.2	8.5 ± 0.8 a	2.4 ± 0.2	7.9 ± 0.8	49.2 ± 1.6
Hairy vetch	6.3 ± 1.2	13.1 ± 1.7	6.1 ± 1.3 ab	1.8 ± 0.3	5.9 ± 1.3	48.7 ± 2.0
Oat	7.4 ± 0.9	14 ± 0.8	5.4 ± 1.0 ab	2.0 ± 0.3	6.3 ± 0.8	52.4 ± 1.7
Rotation+Dry OM						
Carinata	7.4 ± 1.6	14.6 ± 1.2	4.4 ± 1.0 b	1.8 ± 0.2	5.9 ± 0.7	49.2 ± 1.3
Hairy vetch	7.2 ± 0.7	14.3 ± 0.5	6.0 ± 0.6 ab	2.2 ± 0.1	6.7 ± 0.5	48.1 ± 1.8
Oat	6.8 ± 0.6	14.0 ± 0.7	4.8 ± 0.2 ab	1.6 ± 0	5.4 ± 0.3	53.4 ± 1.4

a Mean ± standard error. (n=6)

b Letters within the same plant parameter (column) indicate significant differences between treatments by trial (Tukey's HSD, *P* ≤ 0.05) with ANOVA “***”= *P* ≤ 0.01, “**”= *P* ≤ 0.05. No letters are indicated for *P* > 0.05, denoted as “ns”.

c Rotation+No OM, Rotation+Fresh OM, and Rotation+Dry OM are rotation with no incorporation of shoot organic matter, incorporation of fresh organic matter, and incorporation of dry organic matter, respectively. Fresh roots were incorporated for each treatment.

## Discussion

Crop host status was an important factor in managing RN in this experiment. Oat and fallow were better rotations to lower RN abundances in the soil at winter crop termination (before OM was incorporated) than hairy vetch and carinata. At winter crop termination, only the soil could be sampled, therefore it was not possible to calculate RF, which is necessary to fully determine the host suitability of the crop (Sasser et al., 1984). Nonetheless, this study did reinforce oat as a potential rotation crop to manage RN in the soil since it is a poor host (Asmus et al., 2008; Sandoval-Ruiz and Grabau, 2023). Additionally, consistent with the good host status of hairy vetch (Jones et al., 2006), this crop was not effective at managing RN prior to planting cotton. Carinata did not manage RN as well as expected—no better than hairy vetch at winter crop termination—given it was previously reported as a poor host for RN (Sandoval-Ruiz and Grabau, 2023). Although fallow was one of the best treatments to manage RN in this study, rotations with cover crops have additional benefits compared with fallow, such as decreasing soil erosion and preserving soil and water quality (Adetunji et al., 2020). Additionally, in field conditions, it is difficult and often costly to maintain a clean fallow, since it generally requires repeated tillage, herbicide application, or both to prevent growth of weeds that may maintain or increase nematode populations.

Following incorporation of OM and cotton growth, host status of the previous winter crop was still the most important factor for managing RN, as there were differences among crops—reflecting host status in the first stage of the experiment as discussed above—but not consistently among residue incorporation types. This suggests host status rather than biofumigation was the primary driver of RN management, and the fact that fallow was generally the most effective treatment also supports this hypothesis. It is possible that differences in residue toxicity (Nahar et al., 2006) and volume among non-fallow crop treatments also contributed to RN management and coincided with host status. Residue volume was greater for oat than carinata or hairy vetch, regardless of incorporation type, particularly in Trial 1 when oat was also typically more effective than carinata or hairy vetch at managing RN. Because the chemical composition of residues was not analyzed and the residue volume varied by crop, it is unknown if residue toxicity varied by crop in this experiment and contributed to management. While residue incorporation treatments were intended to help elucidate residue toxicity among crops, this was confounded as residue biomass was generally similar regardless of incorporation type because roots were incorporated for each treatment.

In contrast to this study, crop residue amendments have affected nematode management in some previous studies. For example, applying grasses such as rye and sudangrass reduced *Pratylenchus* more than brassicas such as mustard (Thoden et al., 2011). Similar to this study, nematode management by application of crop OM can also be inconsistent as shown by studies with brassicaceous crops and *M. incognita* (Stapleton and Duncan, 1998) as well as *Brassica juncea* ‘Caliente 199’ and RN (Waisen et al., 2020). Factors such as temperature (Ploeg and Stapleton, 2001; del Carmen Martínez-Ballesta et al., 2013) and OM application rate (Johnson et al., 1999; Zasada and Ferris, 2004) affect biofumigation and variation in these factors between trials may have also affected this study. Tarping with plastic or other methods to seal and solarize the soil may enhance efficacy and consistency of RN management using crop residues (Fourie et al., 2016), but tarping is not practical in cotton production.

As a new crop to the Southeast, investigating carinata for RN management was one of the novel aspects of this experiment. Previous greenhouse experiments have demonstrated that carinata is a poor host for RN (Sandoval-Ruiz and Grabau, 2023), but this experiment more closely represents RN management under field conditions since it also tracked RN after winter crop termination. Based on the results of this experiment, carinata rotation was not as effective as fallow or a common poor RN host winter cover crop (oat) for managing RN, but it was no worse than hairy vetch, a good RN host. Similarly, based on this study, standard carinata residue management practices—incorporating dry residue—are as good as incorporating fresh residues for RN management. More broadly, study results suggest that choosing a non-host is the most important criterion when selecting a winter cover or cash crop for managing RN. Crop incorporation methods or potential biofumigation from crop residues are not as important—at least among crops tested. However, as discussed above, environmental factors may affect RN management using crop rotation or incorporation of crop residues and greenhouse experiments cannot fully reproduce production conditions. Therefore, field research is needed to assess the role of carinata and winter cover crop residue handling in RN management under conditions more relevant for crop production.

Crop rotation and residue incorporation treatments generally did not consistently influence cotton growth. In the first trial, there were some differences among treatments, but in the second trial, treatments did not affect cotton growth. These differences may be related to external conditions such as differences in temperature among trials. In Trial 1, the organic matter material from carinata positively influenced cotton growth. As has been previously reported, organic amendments can increase nutrients and organic carbon and promote growth for the next crop planted (Abawi and Widmer, 2000; Cherr et al., 2006). This was reflected in the results from Trial 1 in that in presence of nematodes, soil amendments from crops such as carinata could provide nutrients to the next cotton crop. A previous report on bananas pointed out that OM can increase plant growth, but this does not necessarily mean a reduction of several nematodes, including RN (Tabarant et al., 2011). In contrast, in Trial 1—where most of the cotton growth differences were seen—while oat helped manage RN in this trial, only cotton root fresh weight was higher with oat than for some of the other treatments, but other plant parameters, such as canopy cover, height and shoot fresh weight, were reduced by this crop. This may be because the high C:N ratio of the winter cereal residues, such as oat, could promote nitrogen immobilization, causing nitrogen deficiency in cotton (Bauer and Reeves, 1999). Another possibility is that allelopathic compounds from the oat residues interfered not only with the nematodes but with some of the plant growth factors, as has been previously reported (Bauer and Reeves, 1999; Grimmer and Masiunas, 2005).

In conclusion, the results from this experiment suggest that the choice of rotational crop affects RN abundance and reproduction. Crop host status is more important for reducing RN population in cotton than the type of OM management used after a rotational crop is grown. Rotational crop and OM incorporation type did not consistently affect cotton growth. Crop rotation using oat can help manage RN pressure better than carinata and hairy vetch, but oat OM amendment incorporation inconsistently, and sometimes negatively, affects cotton growth.
